# Effects of medium-chain fatty acid-rich oils with different free fatty acid contents on fatty acid digestibility, intestinal health and abdominal fat composition in grower-finisher broiler chickens

**DOI:** 10.1016/j.psj.2026.107966

**Published:** 2026-10-14

**Authors:** M. Espinosa-de-los-Monteros-Peñafiel, E. Llauradó-Calero, L. Castillejos, R. Sala, A. Tres, M.D. Soler, A.C. Barroeta

**Affiliations:** a Animal Nutrition and Welfare Service (SNiBA), Department of Animal and Food Sciences, Universitat Autònoma de Barcelona, Bellaterra, 08193, Spain; b Departament de Nutrició, Ciències de l’Alimentació i Gastronomia, Universitat de Barcelona, Santa Coloma de Gramenet, 08921, Spain; c Institut de Recerca en Nutrició i Seguretat Alimentària, Universitat de Barcelona, Santa Coloma de Gramenet, 08921, Spain; d AviFeed Science, Department of Animal Production and Health, Faculty of Veterinary Medicine, Universidad CEU Cardenal Herrera, CEU Universities, Alfara del Patriarca, Valencia, 46115, Spain

**Keywords:** Medium-chain fatty acid, Black soldier fly larvae oil, Palm kernel oil, Palm kernel fatty acid distillate, Lauric acid

## Abstract

This study evaluated the effects of replacing soybean oil (SO) with MCFA-rich oils differing in free fatty acid (FFA) content on fatty acid (FA) digestibility, apparent metabolizable energy (AME), intestinal health, and abdominal fat composition in grower-finisher broilers. Four experimental diets were formulated by replacing 6% of SO with black soldier fly larvae oil (BSFO), palm kernel oil (PKO), or palm kernel fatty acid distillate (PKFAD). At the distal ileum, MCFA-rich diets achieved up to 90% absorption of MCFA, of which more than 96% occurred in the jejunum. However, compared with SO, the inclusion of BSFO and PKO, characterized by high MCFA (36–49%) and low FFA contents (<3%), resulted in lower PUFA digestibility, but similar MUFA and LCSFA utilization (*P <* 0.01). In contrast, the PKFAD diet, with a similar FA composition to PKO but characterized by elevated FFA (68.2%) and LCSFA (38.2%) levels, reduced the digestibility of most FA across intestinal segments and decreased dietary AME (*P <* 0.001). Intestinal morphology and total cecal fermentation were not influenced by dietary treatments. *Lactobacillus* counts were reduced in PKFAD-fed broilers (*P <* 0.01); however, the *Lactobacillus*:Enterobacteriaceae ratio was not affected. Abdominal fat pad weight was not influenced by diet, although MCFA-rich oils modified its FA composition, decreasing PUFA and increasing SFA proportions (both MCFA and LCSFA), resulting in a lower UFA/SFA ratio. Taken together, the inclusion of MCFA-rich oils low in FFA at 6% supports their use as alternative lipid sources in grower–finisher diets by contributing to efficient fatty acid utilization and dietary energy supply, while indicating that FFA levels affect their nutritional value.

## Introduction

Dietary oils are key high-energy ingredients in broiler diets, supplying essential fatty acids (**FA**) and influencing the FA profile of body fat depots (Ferrini et al., 2008). The high cost of dietary energy, together with the price volatility and uncertain supply of conventional feed fat sources such as soybean and palm oils, has increased interest in identifying cost‑effective alternative lipid sources, particularly those derived from food-industry by-products (Varona et al., 2021b).

Among these alternatives, oils rich in medium-chain fatty acids (**MCFA**; C6:0–C12:0) have gained particular interest in poultry nutrition. Lauric acid (C12:0), the predominant MCFA in these oils, has been associated with antimicrobial activity and beneficial effects on intestinal microbiota and gut barrier function in broiler chickens (Baltić et al., 2018; Szabó et al., 2023; Ee et al., 2024). On the one hand, this antimicrobial activity relies on membrane disruption, intracellular acidification, and the downregulation of pathogen invasion genes, suppressing Proteobacteria while promoting short-chain fatty acid (SCFA)-producing Firmicutes, which further creates a favorable luminal environment (Çenesiz and Çiftci, 2020; Xu et al., 2025). On the other hand, although dietary MCFAs have generally shown limited or inconsistent effects on intestinal mucosal structure in healthy broilers, it is described that their potential impact may vary depending on inclusion levels, challenge conditions, or stress environments (Çenesiz and Çiftci, 2020). Moreover, the biological response may differ among MCFA-rich fat sources because they vary not only in MCFA profile, but also in lipid class composition, which may influence lipid digestion, microbial exposure to fatty acids, and ultimately the intestinal response. However, most of this evidence has been generated at low dietary inclusion levels (typically below 2%), at which MCFA-rich oils are used as functional additives rather than as the primary lipid energy source. Consequently, information on the effects of higher dietary inclusion levels of MCFA-rich oils during the grower-finisher phase (21 to 36 d), when digestive function is more fully developed, remains limited. Among MCFA-rich oils, crude palm kernel oil (**PKO**), palm kernel fatty acid distillate (**PKFAD**) and black soldier fly larvae oil (**BSFO**) are characterized by a lauric acid content exceeding 35% of total FA. Both PKO and BSFO are mainly composed of triglycerides (**TAG**), whereas PKFAD, a by-product generated during the physical refining of PKO, contains more than 60% of free fatty acids (**FFA**; Varona et al., 2021a). Previous studies have established that long-chain saturated fatty acids (**LCSFA**, ≥ 14 carbons in length) supplied as FFA show reduced digestibility in starter broiler chickens, whereas FFA absorption and fat utilization improve in older birds (Wiseman et al., 1998; Villaverde et al., 2006; Rodriguez-Sanchez et al., 2019a; Jiménez-Moya et al., 2021b). However, it remains unclear whether these age-related effects associated with FFA also apply to diets in which MCFA are provided in the free form during the grower–finisher phase. Dietary fat sources not only differ in their utilization but also determine the FA profile of body fat depots, which conditions carcass fat quality, its physical properties and its oxidative stability (Sanz, 1999). The abdominal fat pad (**AFP**) is one of the most representative adipose depots in broiler chickens and is commonly used to assess both the extent of fat deposition and the FA composition of body fat reserves (Ferrini et al., 2008). Nevertheless, information on how MCFA-rich fat sources included at high dietary levels affect AFP weight and composition is scarce.

Therefore, it was hypothesized that the nutritive value of MCFA-rich oils depends both on the fatty acid chain length and lipid class composition. Furthermore, at high inclusion levels, these oils may achieve a FA utilization and energy value comparable to those of conventional sources such as soybean oil, while also influencing the fatty acid profile of abdominal fat depots. Given that our previous study focused exclusively on the starter phase (Espinosa-de-los-Monteros-Peñafiel et al., 2026), evaluating these MCFA-rich fat sources and FFA levels in grower–finisher broilers is novel and complementary, providing a comprehensive understanding across the entire growth period. Accordingly, the objective of the present study was to evaluate the effects of including MCFA-rich oils (6%) with different FFA levels in grower–finisher broiler diets (21 to 36 d) on FA digestibility, apparent metabolizable energy (AME), intestinal health (histomorphology, microbial counts, and cecal fermentation), and the FA composition of the abdominal fat pad.

## Materials and methods

### Experimental oils

Four oil sources were used in the present study: soybean oil (**SO**), palm kernel oil (**PKO**) and palm kernel fatty acid distillate (**PKFAD**), provided by LIPSA (Santiga, Spain), and black soldier fly larvae oil (**BSFO**), provided by Protix Ingredients B.V. (Dongen, The Netherlands). The chemical characterization of the four oils followed the methods described by Varona et al. (2021b) for FA composition (obtaining FA methyl esters and determining them by GC-FID), and for moisture, unsaponifiable matter and insoluble impurities (by gravimetry). Lipid classes were determined by GC-MS as described in Espinosa-de-los-Monteros-Peñafiel et al., (2026). The composition of these experimental fats is shown in Table 1.

**Table 1:** Composition of experimental oils included in the grower-finisher diets of broiler chickens.

	Experimental oils^1^			
Item	SO	BSFO	PKO	PKFAD
Fatty acid composition, %				
C6:0	N.D.	N.D.	0.2	0.2
C8:0	N.D.	N.D.	2.6	3.3
C10:0	N.D.	0.8	2.9	3.6
C12:0	N.D.	35.6	43.3	42.7
C14:0	0.1	9.5	17.6	16.7
C16:0	10.8	16.6	9.8	14.7
C16:1 n-7	0.1	2.6	N.D.	<0.1
C18:0	4.5	3.0	2.6	6.7
C18:1 *trans*	0.1	0.1	<0.1	0.1
C18:1 n-9	20.7	15.3	17.6	9.9
C18:1 n-7	1.4	0.4	0.1	0.1
C18:2 n-6	54.0	14.6	2.8	1.9
C18:3 n-3	7.7	1.3	0.1	<0.1
C20:0	0.3	0.1	0.1	0.1
C20:1 n-9	N.D.	0.2	N.D.	<0.1
C22:0	0.3	N.D.	<0.1	N.D.
SFA	16.0	65.6	79.3	87.9
MCFA	N.D.	36.4	49.0	49.7
LCSFA	16.0	29.2	30.2	38.2
MUFA	22.2	18.4	17.8	10.0
PUFA	61.8	15.9	2.9	2.0
Lipid-class composition, %				
TAG	100.0	99.6	98.2	24.1
DAG	N.D.	0.3	1.3	5.7
MAG	N.D.	N.D.	N.D.	2.0
FFA	N.D.	0.1	0.6	68.2
MIU, %	0.99	1.28	0.62	1.11
Moisture	0.01	0.38	0.03	0.11
Impurities	0.21	0.23	0.26	0.08
Unsaponifiable	0.77	0.67	0.33	0.92

Abbreviations: SO: soybean oil; BSFO: black soldier fly larvae oil; PKO: palm kernel oil; PKFAD: palm kernel fatty acid distillate; SFA: saturated fatty acids; MCFA: medium-chain fatty acids (C6:0-C12:0); LCSFA: long-chain saturated fatty acids (≥ C14:0); MUFA: monounsaturated fatty acids; PUFA: polyunsaturated fatty acids; TAG: triglycerides; DAG: diglycerides; MAG: monoglycerides; FFA: free fatty acids; MIU: moisture, impurities and unsaponifiable; N.D.: not detected.

1 All samples were analyzed in duplicate.

### Housing and animals

The trial was conducted at the *Servei de Granges i Camps Experimentals* (Universitat Autònoma de Barcelona; Bellaterra, Barcelona, Spain) and was approved by the institutional Animal Ethics Committee (CEEAH; reference code 4006; DMAH 10167), following European Union Directive 2010/63/EU. A total of 512 female Ross 308 broilers were obtained as one-day-old chicks from a commercial hatchery (Pondex SAU; Lleida, Spain) and allocated to 32 environmentally controlled metabolic cages (16 birds per cage). Cages were randomly assigned to one of four dietary treatments, with eight replicate cages per treatment. The experiment lasted 36 days and consisted of two feeding phases: starter (0 to 21 d of age) and grower (21 to 36 d of age). Feed and water were available *ad libitum* throughout the experimental period, and the room was maintained under the temperature, lighting and humidity program recommended for Ross 308 (Aviagen, 2022). Animals and facilities were inspected at least four times daily. Results from the starter phase have been previously reported in Espinosa-de-los-Monteros-Peñafiel et al. (2026), and the present study focuses on the grower period.

### Experimental design and diets

The grower-finisher diet was based on wheat-soybean meal, fed in mash form, and formulated to meet or exceed FEDNA requirements (2018) and to minimize the basal level of fat (Table 2). Four experimental diets were obtained by adding 6% of the corresponding oil (Table 1) to the basal diet (Table 2), differing exclusively in the fat source: SO (control; 61.8% PUFA, with no detectable (**N.D.**) MCFA or FFA), BSFO (36.4% MCFA and 0.1% FFA), PKO (49.0% MCFA and 0.6% FFA) or PKFAD (49.7% MCFA and 68.2% FFA), the latter obtained as a by-product of the physical refining of crude PKO. The analyzed composition of each of the four experimental diets is shown in Table 3. Ytterbium oxide (Yb₂O₃; 246999, Sigma-Aldrich Chemical Co.; St. Louis, MO) was incorporated into all diets at 50 mg/kg as an inert marker for digestibility determinations. Manufacturing of the basal feed was outsourced to Pinsos Molinet S.A. (Prats de Lluçanès, Barcelona, Spain), whereas the addition of each experimental oil and of the inert marker was performed in-house at the *Servei de Granges i Camps Experimentals* (Universitat Autònoma de Barcelona). The starter diets were previously published in Espinosa-de-los-Monteros-Peñafiel et al. (2026).

**Table 2:** Ingredient composition of the grower-finisher diet (as-fed basis) for broiler chickens.

Ingredients	%
Wheat	44.97
Soybean meal 47%	20.77
Barley	15.00
Wheat middlings	9.68
Experimental oil^1^	6.00
Calcium carbonate	1.17
Dicalcium phosphate	0.62
L-Lysine·HCl	0.38
Sodium chloride	0.28
DL-Methionine	0.28
Vit-Min. premix^2^	0.30
L-Threonine	0.17
L-Valine	0.12
Sodium bicarbonate	0.15
Choline	0.06

1 Soybean oil, Black Soldier Fly larvae oil, Palm Kernel oil, or Palm Kernel Fatty Acid Distillate.

2 Provides, per kg of feed: Vitamin A (retinyl acetate), 9,000 IU; vitamin D3, 3,200 IU; vitamin E (alpha-tocopherol acetate), 60 IU; vitamin B12, 15.0 μg; vitamin B6, 3.0 mg; vitamin K3, 1.99 mg; vitamin B1, 1.99 mg; vitamin B2, 5.01 mg; niacin, 39.99 mg; folic acid, 0.999 mg; biotin, 99 μg; Fe, 50.01 mg; Mn, 99.99 mg; Se, 0.498 mg; Zn (hydroxylated methionine zinc chelate), 39.99 mg; Zn (zinc sulfate monohydrate), 39.99 mg; Cu, 15.24 mg; I, 0.999 mg; 6-Phytase 4a19, 750 FTU; endo-1,4-beta-xylanase, 24,000 BXU.

**Table 3:** Macronutrient and energy content, fatty acid and lipid class composition of the grower-finisher diets fed to broiler chickens (21 to 36 d).

	Grower-Finisher diets			
Item^1^	SO	BSFO	PKO	PKFAD
Macronutrient and energy content, %				
Dry matter	90.12	91.44	90.50	90.42
Crude protein	18.58	18.53	18.56	18.53
SID Methionine⁎	0.50	0.50	0.50	0.50
SID Lysine⁎	1.00	1.00	1.00	1.00
Ether extract	7.64	7.31	7.51	7.65
Crude fiber	3.81	3.90	3.87	3.86
Ash	4.72	4.94	4.78	4.79
Calcium*	0.82	0.82	0.82	0.82
Phosphorus*	0.51	0.51	0.51	0.51
Digestible phosphorus*	0.40	0.40	0.40	0.40
Chloride*	0.30	0.30	0.30	0.30
Sodium*	0.20	0.20	0.20	0.20
Gross energy, kcal/kg	4,300	4,258	4,248	4,239
Fatty acid composition, %				
C8:0	N.D.	N.D.	2.3	2.8
C10:0	N.D.	0.6	2.2	2.6
C12:0	1.1	27.2	32.7	32.8
C14:0	N.D.	6.4	11.3	10.6
C16:0	12.7	16.4	11.8	14.7
C18:0	3.9	2.9	2.6	5.2
C18:1 n-9	18.9	15.0	16.2	11.2
C18:1 n-7	1.3	0.6	0.4	0.4
C18:2 n-6	54.3	26.6	18.8	18.1
C18:3 n-3	6.9	2.4	1.6	1.6
C20:0	0.3	N.D.	N.D.	N.D.
C20:1 n-9	0.3	0.2	0.3	N.D.
C22:0	0.3	N.D.	N.D.	N.D.
Minor fatty acids %	N.D.	1.7	N.D.	N.D.
SFA	18.3	53.5	62.7	68.7
MCFA	1.1	27.9	37.2	38.2
LCSFA	17.2	25.6	25.5	30.5
MUFA	20.5	17.5	16.9	11.6
PUFA	61.2	29.0	20.4	19.7
Lipid-class composition, %				
TAG	72.0	73.9	75.4	23.3
DAG	6.9	6.3	6.9	5.6
MAG	0.1	0.1	0.1	0.1
FFA	21.0	19.8	17.6	69.0

Abbreviations: SO: soybean oil 6%; BSFO: black soldier fly larvae oil 6%; PKO: palm kernel oil 6%; PKFAD: palm kernel fatty acid distillate 6%; MCFA: medium-chain fatty acids (C6:0-C12:0); LCSFA: long-chain saturated fatty acids (≥ C14:0); SFA: saturated fatty acids; MUFA: monounsaturated fatty acids; PUFA: polyunsaturated fatty acids. TAG: triglycerides; DAG: diglycerides; MAG: monoglycerides; FFA: free fatty acids; N.D.: not detected.

⁎ Calculated composition.

1 All samples were analyzed in duplicate.

### Controls and sampling

Individual body weight (**BW**) and feed intake per cage were recorded at 21 and 36 days of age to calculate average daily feed intake (**ADFI**), average daily gain (**ADG**), and feed conversion ratio (**FCR**). Mortality was recorded, and individual weights were taken to adjust these variables. At 36 d, excreta were collected from the cage trays for the digestibility balance and AME determination. Two birds per cage were then selected based on the average BW of the replicate, stunned using an electrical stunner (Ref. 105523; FAF, Saint-Sernin-sur-Rance, France), and then, euthanized. The abdominal fat pad of both birds (from the proventriculus to the cloaca) was excised and weighed individually to express its weight relative to live BW; samples were subsequently homogenized, pooled by replicate, and stored at −20°C until FA composition analysis. Detailed sampling procedures for digesta and tissue from the distal jejunum and distal ileum, and for cecal content, including sample processing and storage, were identical to those described in Espinosa-de-los-Monteros-Peñafiel et al. (2026). Briefly, from one of the two selected birds, jejunal and ileal segments were excised and divided into two equal portions, using the distal portions for digesta and tissue sampling. For digestibility assessment, digesta from distal segments were collected, freeze-dried, and processed for analysis. For intestinal health evaluation, tissue samples from the same regions were fixed for histomorphology, whereas digesta were used for microbiological analysis, and cecal content was collected for volatile fatty acids (VFA) determination.

### Chemical analysis

Analytical determinations of feed, digesta, excreta were performed as described in Espinosa-de-los-Monteros-Peñafiel et al. (2026).

Diets were analyzed for proximate composition according to AOAC (2023), and gross energy was determined by bomb calorimetry. Yb₂O₃ was used as an inert marker and quantified in diets, digesta, and excreta by ICP-OES (AOAC 984.27).

Lipid classes were determined by GC-MS as described in Espinosa-de-los-Monteros-Peñafiel et al., (2026). Fatty acid content in diets, digesta, and excreta was quantified by direct transesterification (Sukhija and Palmquist, 1988), using C19:0 as an internal standard. On the other hand, the FA composition of the abdominal fat pad was obtained following the methodology described by Carrapiso et al. (2000). The resulting extracts were analyzed using a gas chromatograph equipped with a flame ionization detector (**FID**; HP 6890, Agilent Technologies, Waldbronn, Germany) under the conditions previously described by Cortinas et al. (2004). The FA methyl esters were identified by comparison of retention times with those of authentic standards (Supelco 37 Component FAME Mix, Bellefonte, PA, USA), and individual FA were quantified from external calibration curves prepared with the same standards.

### Intestinal health evaluation

Intestinal health parameters, including histomorphology, microbial counts, and VFA levels, were analyzed following the methods previously reported in Espinosa-de-los-Monteros-Peñafiel et al. (2026).

Histomorphological analysis was performed on paraffin-embedded intestinal sections (4 μm) stained with hematoxylin and eosin. Villus height (**Vh**) and crypt depth (**Cd**) were measured on digitized slides, with 30 measurements per bird used to calculate the villus-to-crypt ratio (**Vh:Cd**).

Microbial populations were quantified by culture-based methods using selective media (MRS and MacConkey agar) under appropriate incubation conditions, and results were expressed as CFU/g of digesta.

Cecal VFA were analyzed by GC-FID after sample homogenization, centrifugation, acidification, and standard preparation procedures.

### Calculations

The apparent digestibility of FA was calculated as follows:

$$\%\;\mathrm{Apparent}\;\mathrm{digestibility}\;\mathrm{of}\;X=\left\{1-\left[\left(X_{f}/M_{f}\right)/\left(X_{d}/M_{d}\right)\right]\right\}\times 100$$

where X represents the level of a specific FA found in excreta or digesta (f), and in diet (d), whereas M indicates the concentration of the inert marker in feces or digesta (f) and in diet (d). Apparent metabolizable energy (AME) of each diet was calculated from the gross energy of the diet and of the excreta, corrected using the inert marker, and expressed on an as-fed basis (kcal/kg).

### Statistical analysis

Data normality was assessed by Shapiro-Wilk test in the R statistical package (version 4.2.2). All statistical analyses were conducted using one-way ANOVA in R software, with diet considered as the primary factor for all variables. The experimental unit was the cage, with 6 replicates per treatment for digestibility and 8 replicates for intestinal health assessment, growth performance, and abdominal fat analyses, and differences between means were evaluated using Tukey's adjustment for multiple comparisons. The results shown in the tables represent least squares means. Significance was established at *P* ≤ 0.05 for all statistical analyses.

## Results

### Characterization of experimental oils and diets

The composition of the experimental oils is shown in Table 1. Regarding the FA composition, SO had the highest PUFA content (61.8%), mainly linoleic acid (C18:2 n-6), and the lowest content of saturated fatty acids (**SFA**; 16.0%). The three MCFA-rich oils (BSFO, PKO and PKFAD) contained > 65% SFA, of which > 35% corresponded to MCFA, with lauric acid (C12:0) as the main FA. The BSFO showed a lower C12:0 content (35.6%) and a higher PUFA content (15.9%) than the palm kernel-derived oils (PKO, C12:0: 43.3%, PUFA: 2.9%; PKFAD, C12:0: 42.7%, PUFA: 2.0%). Among all the experimental oils, PKFAD had the lowest MUFA (10%), and the highest LCSFA (38.2%) and FFA content (PKFAD: 68.2%; SO: N.D.; BSFO: 0.1%; PKO: 0.6%). The total moisture, impurities, and unsaponifiable content (**MIU**) of the experimental oils were below 1.3%.

The composition of the experimental diets is shown in Table 3. Gross energy and all macronutrient contents were similar among dietary treatments. The FA composition reflected that of the corresponding experimental oils, with the diets containing BSFO and palm kernel-derived oils (PKO and PKFAD) being richer in MCFA, whereas the SO diet was richer in PUFA. In terms of dietary lipid-class composition, TAG were the main lipid class in SO, BSFO and PKO diets (72.0–75.4%), whereas the PKFAD diet contained a much lower TAG proportion (23.3%) and was dominated by FFA (69.0%).

### Growth performance

The effects of the experimental diets on growth performance are presented in Table 4. The BW at 36 d was lower in PKFAD-fed broilers than in those fed SO, BSFO or PKO (*P <* 0.001), with no differences among the latter three treatments. The ADG showed a numerical decrease in PKFAD-fed broilers, with the effect approaching significance (*P =* 0.053), whereas no effect of dietary treatment was observed on ADFI or FCR **(***P* > 0.05).

**Table 4:** Effect of the experimental diets on growth performance in broiler chickens (21 to 36 d).

	Dietary treatments^1^					
Item^2^	SO	BSFO	PKO	PKFAD	SEM	*P*-value
BW at 36 d, g	2,259 a	2,238 a	2,200 a	2,058 b	28.1	<0.001
From 21 to 36 d						
ADFI, g/d	140	140	137	134	3.16	0.526
ADG, g/d	92.2	91.3	91.0	86.6	1.46	0.053
FCR, g/g	1.52	1.55	1.51	1.54	0.026	0.663

1 Abbreviations: SO: soybean oil; BSFO: black soldier fly larvae oil; PKO: palm kernel oil; PKFAD: palm kernel fatty acid distillate.

2 Values of ADFI and ADG are expressed as-fed basis; BW: body weight; ADFI: average daily feed intake; ADG: average daily gain; FCR: feed conversion ratio.

a-b Values within a row with different superscripts differ significantly at *P* ≤ 0.05 according to one-way ANOVA (n = 8 replicates per treatment; 4 chickens per replicate); SEM: standard error of the mean.

### Digestibility balance

The apparent digestibility of FA at the distal jejunum, distal ileum and total tract is presented in Table 5. For data presentation, SFA were subdivided into MCFA (C6:0, C8:0, C10:0 and C12:0) and LCSFA (C14:0, C16:0 and C18:0). Unsaturated FA (UFA) comprised MUFA (mainly oleic acid, C18:1 n-9) and PUFA (mainly linoleic acid, C18:2 n-6, and α-linolenic acid, C18:3 n-3).

**Table 5:** Effect of experimental diets on apparent fatty acid digestibility at the distal jejunum, distal ileum, and total tract in broiler chickens at 36 d of age.

	Dietary treatments^1^					
Item	SO	BSFO	PKO	PKFAD	SEM	*P*-value
Distal jejunum						
C8:0	N.D.	N.D.	99.5	98.5	0.67	0.259
C10:0	N.D.	100.0 ^a^	99.4 ^a^	94.7 ^b^	0.37	<0.001
C12:0	37.6 ^b^	93.0 ^a^	93.2 ^a^	86.9 ^a^	2.71	<0.001
C14:0	N.D.	93.0 ^a^	93.7 ^a^	82.5 ^b^	0.89	<0.001
C16:0	72.7	74.1	68.5	68.7	2.36	0.190
C18:0	54.8	53.0	44.9	62.2	5.72	0.199
C18:1 n-9	84.0 ^a^	81.8 ^a^	81.7 ^a^	68.9 ^b^	2.81	0.002
C18:2 n-6	84.9 ^a^	70.6 ^ab^	60.2 ^b^	54.1 ^b^	5.04	0.001
C18:3 n-3	91.0 ^a^	78.7 ^ab^	68.3 ^b^	66.3 ^b^	3.56	<0.001
TFA	82.9 ^a^	80.6 ^a^	80.5 ^a^	70.9 ^b^	2.51	0.005
MCFA	37.6 ^b^	93.5 ^a^	94.3 ^a^	87.4 ^a^	2.76	<0.001
LCSFA	79.1 ^a^	74.6 ^ab^	73.9 ^ab^	63.5 ^b^	3.33	0.014
MUFA	83.9 ^a^	81.5 ^a^	82.7 ^a^	64.0 ^b^	3.03	<0.001
PUFA	85.4 ^a^	73.4 ^ab^	60.6 ^bc^	54.0 ^c^	5.00	0.001
Distal ileum						
C8:0	N.D.	N.D.	100.0 ^a^	97.2 ^b^	0.08	<0.001
C10:0	N.D.	100.0 ^a^	100.0 ^a^	95.7 ^b^	0.27	<0.001
C12:0	41.5 ^c^	94.6 ^a^	95.3 ^a^	88.8 ^b^	1.43	<0.001
C14:0	N.D.	94.7 ^a^	95.9 ^a^	85.1 ^b^	1.06	<0.001
C16:0	82.0 ^a^	79.3 ^ab^	78.9 ^ab^	74.1 ^b^	1.47	0.005
C18:0	82.7	76.5	78.4	74.6	2.30	0.093
C18:1 n-9	88.8 ^a^	80.2 ^b^	84.6 ^ab^	75.0 ^c^	1.22	<0.001
C18:2 n-6	92.2 ^a^	78.6 ^b^	74.6 ^b^	73.6 ^b^	2.08	<0.001
C18:3 n-3	95.6 ^a^	84.1 ^b^	79.3 ^b^	79.1 ^b^	1.58	<0.001
TFA	89.5 ^a^	84.4 ^bc^	87.2 ^ab^	81.3 ^c^	1.06	<0.001
MCFA	41.5 ^c^	94.7 ^a^	95.9 ^a^	89.9 ^b^	1.37	<0.001
LCSFA	82.7 ^ab^	82.2 ^ab^	86.3 ^a^	77.9 ^b^	1.54	0.004
MUFA	88.7 ^a^	81.1 ^ab^	84.0 ^ab^	73.8 ^b^	1.20	<0.001
PUFA	92.6 ^a^	79.0 ^b^	74.9 ^b^	74.0 ^b^	2.07	<0.001
Excreta						
C8:0	N.D.	N.D.	99.1 ^a^	97.8 ^b^	0.38	0.017
C10:0	N.D.	100.0 ^a^	97.8 ^b^	96.2 ^c^	0.36	<0.001
C12:0	86.2 ^b^	96.5 ^a^	95.2 ^a^	91.7 ^a^	1.43	<0.001
C14:0	N.D.	95.2 ^a^	92.9 ^a^	86.8 ^b^	1.02	<0.001
C16:0	85.3 ^a^	83.1 ^ab^	81.4 ^b^	77.8 ^c^	0.73	<0.001
C18:0	89.4 ^a^	82.1 ^b^	82.3 ^b^	79.6 ^b^	0.86	<0.001
C18:1 n-9	90.2 ^a^	85.8 ^b^	86.9 ^b^	79.2 ^c^	0.44	<0.001
C18:2 n-6	93.0 ^a^	82.9 ^b^	80.9 ^b^	77.5 ^c^	0.78	<0.001
C18:3 n-3	95.6 ^a^	85.9 ^b^	82.8 ^c^	80.4 ^c^	0.78	<0.001
TFA	91.2 ^a^	88.4 ^b^	88.4 ^b^	84.7 ^c^	0.55	<0.001
MCFA	86.2 ^b^	96.6 ^a^	95.6 ^a^	92.4 ^a^	1.41	<0.001
LCSFA	85.8 ^a^	86.5 ^a^	86.6 ^a^	81.0 ^b^	0.69	<0.001
MUFA	89.9 ^a^	85.9 ^b^	86.2 ^b^	77.9 ^c^	0.44	<0.001
PUFA	93.3 ^a^	83.2 ^b^	81.0 ^b^	77.7 ^c^	0.77	<0.001
AME, kcal/kg						
	3,323 ^a^	3,272 ^a^	3,321 ^a^	3,177 ^b^	15.7	<0.001

1 Values are pooled means of 6 replicates (1 chicken per replicate) from chickens fed diets supplemented with 6% of: soybean oil (SO); black soldier fly larvae oil (BSFO); palm kernel oil (PKO); palm kernel fatty acid distillate (PKFAD). TFA: total fatty acids, MCFA: medium-chain fatty acids, LCSFA: long-chain saturated fatty acids, MUFA: monounsaturated fatty acids, PUFA: polyunsaturated fatty acids. SEM: standard error of the mean. ^a-c^ Values within a row with different superscripts differ significantly at *P* ≤ 0.05. N.D.: not detected.

The digestibility of the individual FA in the ileum is shown in Fig. 1. Although the jejunum remained a key site of absorption, distal ileal digestibility was selected as it reflects the final stage of fat absorption. Among the SFA, MCFA showed the highest digestibility values, with C12:0 reaching values around 95% in birds fed BSFO and PKO. These values were similar to, or even higher than, those observed for C18:2 n-6 in SO-fed birds (92%). In contrast, the LCSFA C16:0 and C18:0 presented the lowest SFA digestibility across treatments, with the most limited absorption observed in broilers fed the PKFAD diet (C16:0: 74%; C18:0: 75%), which contained high levels of both FFA and LCSFA.

**Fig. 1:**
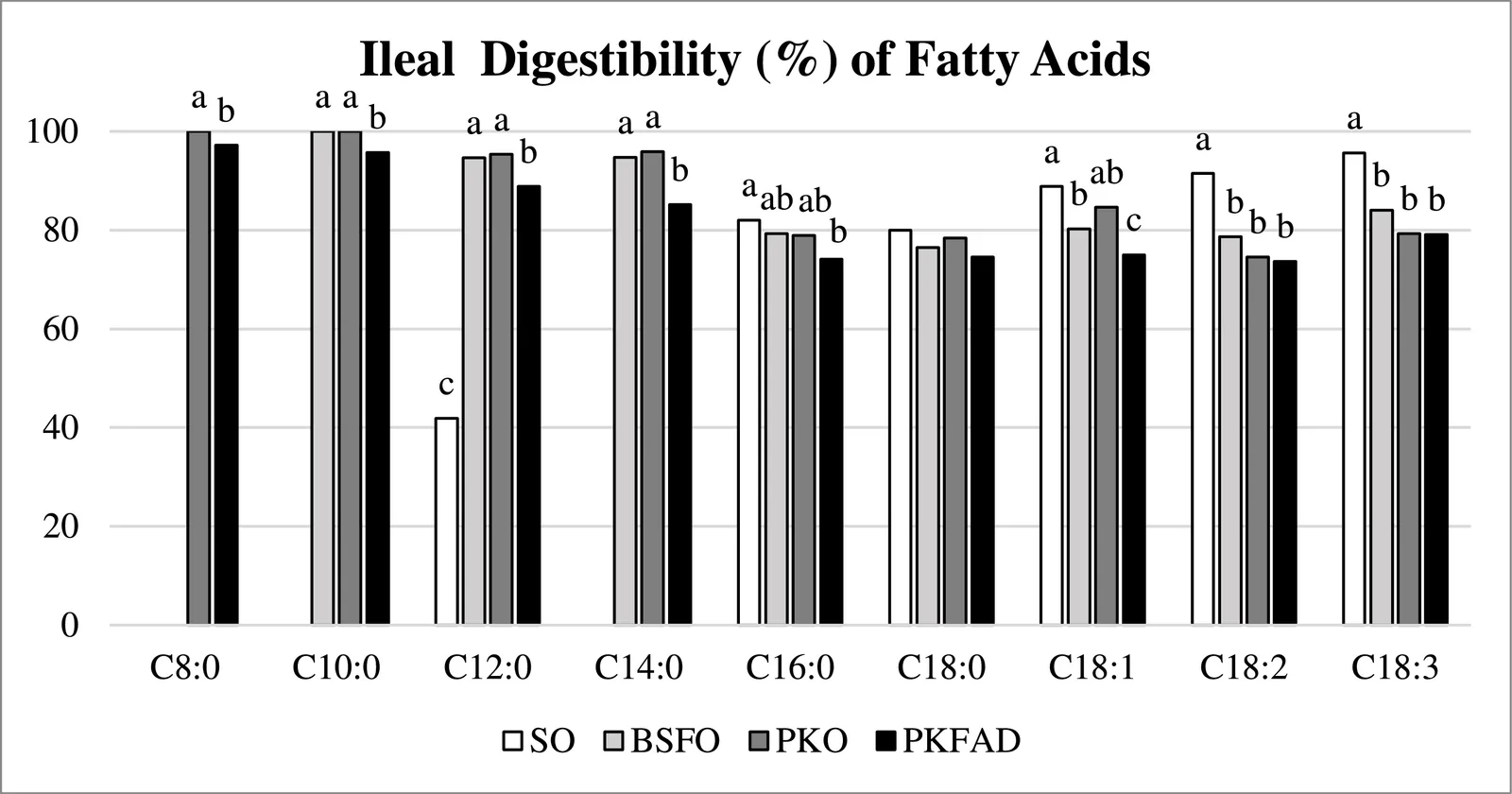
Apparent digestibility (%) of individual fatty acids in the distal ileum of 36 d of age broiler chickens fed different diets with 6% of: soybean oil (SO), black soldier fly larvae oil (BSFO), palm kernel oil (PKO), or palm kernel fatty acid distillate (PKFAD). Values are means of 6 replicates per diet (1 chicken per replicate). Within each fatty acid, bars with different superscripts (a-c) differ (*P* < 0.01). See Table 5 for detailed *P-*values.

Broilers fed BSFO, PKO and PKFAD showed higher MCFA digestibility, particularly for lauric acid (C12:0), than those fed SO at all sampling sites (*P <* 0.001). Among the MCFA-rich diets, BSFO and PKO did not differ in MCFA digestibility at any segment or in excreta, whereas PKFAD showed lower C12:0 digestibility at the distal ileum than broilers fed BSFO or PKO (BSFO: 94.6% and PKO: 95.3% *vs.* PKFAD: 88.8%; *P <* 0.001).

Within LCSFA, C14:0 digestibility was high and similar in BSFO- and PKO-fed broilers across all intestinal segments (93.0 to 95.9%) and was lower in those fed PKFAD (82.5 to 86.8%; *P* < 0.001). Digestibility of C16:0 and C18:0 did not differ among treatments at the distal jejunum. Finally, and considering total LCSFA digestibility in the jejunum, ileum, and excreta, the lowest values were observed in broilers fed PKFAD (*P* < 0.05). Across all sampling sites, the lowest MUFA digestibility was in PKFAD-fed broilers, with no differences among the remaining treatments. The PUFA digestibility was highest in birds fed SO across all intestinal segments (*P* < 0.001), with no differences among BSFO, PKO and PKFAD at the distal ileum or in the total tract.

Total FA (**TFA**) digestibility did not differ among SO, BSFO and PKO at the distal jejunum, whereas it was lower in PKFAD‑fed broilers (*P* = 0.005). At the distal ileum, SO showed the highest TFA digestibility (89.5%), followed by PKO (87.2%), BSFO (84.4%) and PKFAD (81.3%; *P* < 0.001). Dietary AME did not differ among birds fed SO, BSFO or PKO and was lower in those fed PKFAD (SO: 3,323 kcal/kg, BSFO: 3,272 kcal/kg and PKO: 3,321 kcal/kg *vs.* PKFAD: 3,177 kcal/kg; *P* < 0.001).

The relative contribution of the jejunum and ileum to FA absorption, expressed as a percentage of total ileal absorption (considered 100%), is shown in Fig. 2. Overall, FA utilization occurred predominantly in the jejunum across all classes. Across all dietary treatments, MCFA showed the highest jejunal contribution (91–98%), followed by MUFA (86–97%). Notably, broilers fed the PKFAD diet showed the highest ileal contribution, particularly for PUFA (approximately 28%), LCSFA (19%) and MUFA (14%), reflecting a shift toward more distal absorption.

**Fig. 2:**
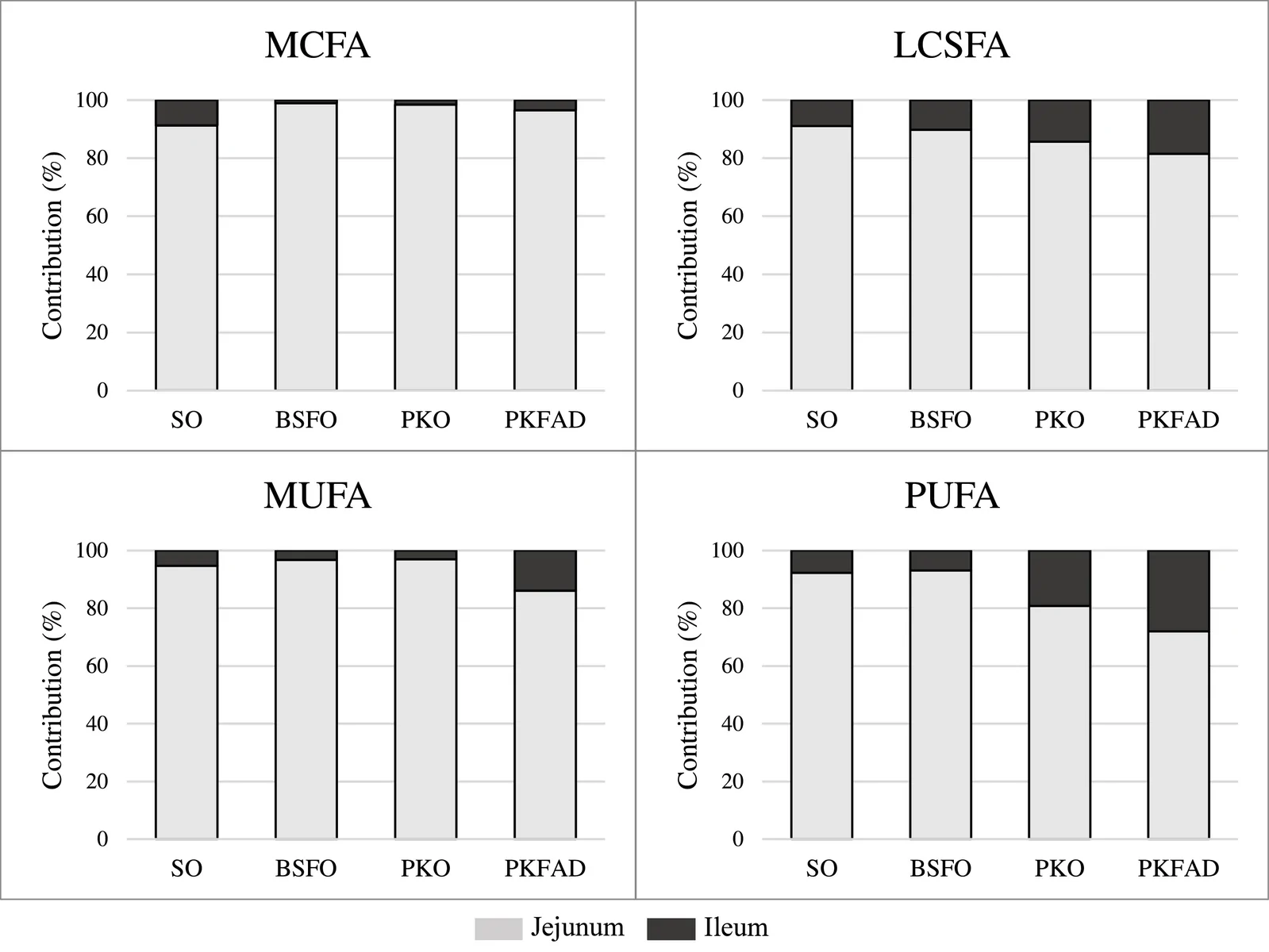
Contribution of the jejunum and ileum to the apparent digestibility of FA, expressed as a percentage of total absorption at distal ileum level in 36 d of age broiler chickens fed diets with 6% of: soybean oil (SO), black soldier fly larvae oil (BSFO), palm kernel oil (PKO), or palm kernel fatty acid distillate (PKFAD). MCFA: medium-chain fatty acids; LCSFA: long-chain saturated fatty acids; MUFA: monounsaturated fatty acids; PUFA: polyunsaturated fatty acids. Values are means of 6 replicates per diet (1 chicken per replicate).

### Histomorphology of jejunum and ileum

Intestinal histomorphology results are summarized in Table 6. At the distal jejunum, no differences were observed among treatments for Vh, Cd or the Vh:Cd ratio (*P* > 0.05). At the distal ileum, no differences were observed among treatments for Cd or the Vh:Cd ratio (*P* > 0.05); however, broilers fed SO showed higher Vh than those fed PKO (*P* = 0.040).

**Table 6:** Effect of the experimental diets on histomorphology of the distal jejunum and distal ileum in broiler chickens at 36 d of age.

	Dietary treatments^1^					
	SO	BSFO	PKO	PKFAD	SEM	*P*-value
Jejunum						
Vh, μm	1,900	1,915	1,901	1,799	75.3	0.631
Cd, μm	344	332	346	319	12.3	0.378
Vh:Cd ratio	5.54	5.54	5.38	5.65	0.16	0.662
Ileum						
Vh, μm	1,266 ^a^	1,178 ^ab^	1,082 ^b^	1,155 ^ab^	45.3	0.040
Cd, μm	354	332	336	328	8.87	0.099
Vh:Cd ratio	3.59	3.56	3.24	3.59	0.18	0.288

1 Values are pooled means of 8 replicates per diet (1 chicken per replicate) from chickens fed diets supplemented with 6% of soybean oil (SO), black soldier fly larvae oil (BSFO), palm kernel oil (PKO), and palm kernel fatty acid distillate (PKFAD). Villus height (Vh), crypt depth (Cd). SEM: standard error of the mean.^a-b^ Values within a row with different superscripts differ significantly at *P* ≤ 0.05 according to one-way ANOVA.

### Microbiological counting

Microbiological counts obtained under the experimental treatments are reported in Table 7.

**Table 7:** Effect of experimental diets on the microbiological counting of the distal jejunum and ileum in broiler chickens at 36 d of age.

	Dietary treatments^1^					
	SO	BSFO	PKO	PKFAD	SEM	*P*-value
**Jejunum**						
*Lactobacillus*, log CFU/g	7.35 ^a^	6.84 ^ab^	7.89 ^a^	6.13 ^b^	0.34	0.004
Enterobacteriaceae, log CFU/g	3.92	3.92	4.56	4.03	0.41	0.642
Total coliforms, log CFU/g	3.86	3.92	4.66	4.15	0.41	0.503
*Escherichia coli*, log CFU/g	4.03	3.68	4.22	4.19	0.40	0.739
*Lactobacillus:*Enterobacteriaceae ratio	1.77	1.88	1.75	1.75	0.21	0.961
**Ileum**						
*Lactobacillus*, log CFU/g	6.91 ^ab^	7.76 ^a^	7.84 ^a^	5.67 ^b^	0.39	0.001
Enterobacteriaceae, log CFU/g	4.34	5.24	5.27	4.31	0.63	0.544
Total coliforms, log CFU/g	4.34	5.24	5.36	4.31	0.65	0.525
*Escherichia coli*, log CFU/g	4.33	5.62	5.79	4.54	0.67	0.210
*Lactobacillus:*Enterobacteriaceae ratio	1.60	1.82	1.69	1.37	0.26	0.638

1 Values are pooled means of 8 replicates per diet (1 chicken per replicate) from broilers fed diets supplemented with 6% soybean oil (SO), black soldier fly larvae oil (BSFO), palm kernel oil (PKO), or palm kernel fatty acid distillate (PKFAD). SEM: standard error of the mean. ^a-b^ Values within a row with different superscripts differ significantly at *P* ≤ 0.05 according to one-way ANOVA.

Differences among treatments were observed only for *Lactobacillus* counts. At the distal jejunum, *Lactobacillus* counts were lower in PKFAD-fed broilers than in those fed SO or PKO (*P* = 0.004). At the distal ileum, *Lactobacillus* counts were lower in PKFAD-fed broilers than in those fed BSFO or PKO (*P* = 0.001). No differences were observed among treatments for *Enterobacteriaceae*, total coliforms, *Escherichia coli* counts or *Lactobacillus:Enterobacteriaceae* ratio at either intestinal segment (*P* > 0.05).

### Cecal VFA content

Cecal fermentation data are presented in Table 8. No differences were observed among treatments for total VFA concentration or for the proportions of acetate, butyrate, valerate or branched-chain fatty acids (**BCFA**; *P* > 0.05). The propionate content approached significance (*P* = 0.063), with numerically higher values in birds fed SO and BSFO than in those fed PKO and PKFAD.

**Table 8:** Effect of experimental diets on fermentation products in the cecal content of broiler chickens at 36 d of age.

	Dietary treatments^1^					
Volatile fatty acids	SO	BSFO	PKO	PKFAD	SEM	*P*-value
Total SCFA, μmol/g	96.1	93.4	84.1	87.2	9.01	0.770
Acetate, %	78.9	80.3	82.2	82.3	1.26	0.169
Propionate, %	3.12	3.27	2.26	2.26	0.33	0.063
Butyrate, %	16.8	15.3	15.3	14.6	1.09	0.518
BCFA, %	0.454	0.388	0.325	0.278	0.029	0.670

1 Values are pooled means of 8 replicates per diet (1 chicken per replicate) from broilers fed diets supplemented with 6% of soybean oil (SO), black soldier fly larvae oil (BSFO), palm kernel oil (PKO), and palm kernel fatty acid distillate (PKFAD); short chain fatty acids (SCFA); branched chain fatty acids (BCFA). SEM: standard error of the mean. ^a-b^ Values within a row with different superscripts differ significantly at *P* ≤ 0.05 according to one-way ANOVA.

### Fatty acid composition of abdominal fat pad

The FA composition and relative weight of the AFP are presented in Table 9. The AFP weight (% BW) and the total FA content (mg/g) did not differ among treatments (*P* > 0.05). With respect to the FA profile, broilers fed the SO diet showed the highest PUFA content and the lowest SFA proportion, including both MCFA and LCSFA, compared with the remaining treatments (*P <* 0.001). Among the MCFA-rich diets, no differences were found in total MUFA content. However, the BSFO treatment showed higher PUFA levels, particularly linoleic (C18:2 n-6) and α-linolenic (C18:3 n-3) acids, and lower total SFA content compared with PKO and PKFAD (*P <* 0.001).

**Table 9:** Effect of experimental diets on fatty acid composition (%) of abdominal fat pad of broiler chickens at 36 d of age.

		Dietary treatments^1^				
Item	SO	BSFO	PKO	PKFAD	SEM	*P*-value
Abdominal fat, % BW	1.77	2.00	1.76	1.92	0.08	0.102
Total FA, mg/g	746	736	703	802	61.2	0.716
SFA	26.0 ^c^	44.2 ^b^	48.4 ^a^	48.5 ^a^	0.45	< 0.001
MCFA	0.1 ^c^	12.0 ^b^	15.2 ^a^	12.9 ^b^	0.45	< 0.001
LCSFA	25.9 ^c^	32.5 ^b^	33.6 ^b^	35.7 ^a^	0.34	< 0.001
MUFA	37.8 ^b^	40.5 ^a^	42.3 ^a^	42.0 ^a^	0.70	< 0.001
PUFA	36.5 ^a^	15.3 ^b^	9.5 ^c^	9.5 ^c^	0.49	< 0.001
UFA/SFA	2.9 ^a^	1.3 ^b^	1.1 ^c^	1.1 ^c^	0.03	< 0.001
C8:0	N.D.	0.04 ^c^	0.12 ^b^	0.14 ^a^	0.01	< 0.001
C10:0	N.D.	0.2 ^b^	0.5 ^a^	0.5 ^a^	0.02	< 0.001
C12:0	0.1 ^c^	11.8 ^b^	14.6 ^a^	12.2 ^b^	0.43	< 0.001
C14:0	0.4 ^d^	5.1 ^c^	8.2 ^a^	7.1 ^b^	0.17	< 0.001
C14:1	0.1 ^c^	0.8 ^b^	1.1 ^a^	1.1 ^a^	0.04	< 0.001
C16:0	19.6 ^c^	22.7 ^a^	21.0 ^b^	23.2 ^a^	0.36	< 0.001
C16:1 n-7	3.8 ^b^	6.4 ^a^	5.8 ^a^	6.5 ^a^	0.29	< 0.001
C18:0	5.3 ^a^	4.4 ^b^	4.2 ^b^	5.1 ^a^	0.13	< 0.001
C18:1 n-9	31.6	31.1	32.2	31.8	0.47	0.413
C18:1 n-7	1.9 ^b^	1.9 ^b^	2.0 ^ab^	2.1 ^a^	0.06	0.004
C18:2 n-6	31.9 ^a^	13.7 ^b^	8.6 ^c^	8.6 ^c^	0.43	< 0.001
C18:3 n-3	4.0 ^a^	1.1 ^b^	0.6 ^c^	0.7 ^c^	0.05	< 0.001

1 Values are pooled means of 8 replicates per diet (2 chickens per replicate) from broilers fed diets supplemented with 6% of soybean oil (SO), black soldier fly larvae oil (BSFO), palm kernel oil (PKO), and palm kernel fatty acid distillate (PKFAD). SFA: saturated fatty acids; MCFA: medium-chain fatty acids; LCSFA: long-chain saturated fatty acids; MUFA: monounsaturated fatty acids; PUFA: polyunsaturated fatty acids; UFA: unsaturated fatty acids; SEM: standard error of the mean. Abdominal fat (%) is expressed as a percentage of live body weight. ^a-d^ Values within a row with different superscripts differ significantly at *P* ≤ 0.05. N.D.: not detected.

When comparing PKO and PKFAD, broilers fed PKFAD showed a lower MCFA content, mainly due to a lower proportion of lauric acid (C12:0), and a higher LCSFA content, specifically palmitic (C16:0) and stearic (C18:0) acids, than broilers fed PKO (*P <* 0.001).

## Discussion

### Growth performance

In the present study, the replacement of SO by BSFO or PKO did not modify growth performance in grower–finisher broilers, with no significant differences in BW, ADFI, ADG, or FCR among these treatments. These results are consistent with previous reports showing that the dietary inclusion of BSFO does not impair broiler growth performance from 28 d of age onward (Schiavone et al., 2017; Kim et al., 2020; Chen et al., 2022), and with studies using coconut oil, which has a FA profile similar to that of PKO (Wang et al., 2015; Kim et al., 2020).

In contrast, broilers fed PKFAD showed the lowest BW at 36 d, representing reductions of 8.90% relative to SO, 8.04% relative to BSFO, and 6.45% relative to PKO. This was associated with a lower ADG that approached significance, although FCR was not significantly affected. This response could be attributed to a reduction in dietary AME (PKFAD: 3,177 vs. SO: 3,323, PKO: 3,321, and BSFO: 3,272 kcal/kg; P<0.001). In turn, this was accompanied by reduced digestibility of the LCSFA and MUFA fractions in PKFAD-fed birds, which aligns with the limited utilization of LCSFA supplied as FFA previously reported in broilers (Wiseman and Salvador, 1991; Rodriguez-Sanchez et al., 2019a). Overall, these results indicate that MCFA-rich oils evaluated may serve as viable alternatives to SO in grower–finisher broiler diets without compromising FCR. In addition, crude MCFA-rich oils with low FFA content achieved comparable growth performance and dietary energy utilization to SO.

### Fatty acid digestibility and apparent metabolizable energy

The present results confirm that FA digestibility in broilers is primarily determined by the physicochemical characteristics of the FA, in agreement with classical concepts in lipid nutrition (Krogdahl, 1985; Wiseman and Blanch, 1994). At the distal ileum, which represents the final stage of FA absorption, MCFA consistently exhibited the highest digestibility coefficients, followed by PUFA and MUFA, whereas LCSFA, particularly C16:0 and C18:0, remained the least digestible. This pattern was already observed in starter birds and is consistent with previous studies in broilers (Tancharoenrat et al., 2014; Rodriguez-Sanchez et al., 2019a; Espinosa-de-los-Monteros-Peñafiel et al., 2026).

An age-related improvement in FA utilization was observed when comparing the present results with those obtained during the starter phase (Espinosa-de-los-Monteros-Peñafiel et al., 2026). In particular, the digestibility of lauric acid (C12:0) increased from approximately 89–90% to above 93–95% in grower–finisher broilers. This improvement can be attributed to the progressive development of the digestive system, including increased bile salt secretion, pancreatic lipase activity, and micellar solubilization capacity (Leeson and Atteh, 1995; Ravindran et al., 2016). However, the digestibility pattern among individual FA remained unchanged, confirming that chain length and degree of unsaturation are the primary determinants of absorption efficiency regardless of age.

The high digestibility of MCFA observed in broilers fed MCFA-rich oils at the grower–finisher stage (90-96% at distal ileum) is consistent with the known absorption mechanisms of these FA. Because MCFA are more water-soluble than LCSFA, they are likely absorbed more rapidly, a process that would occur partly via passive diffusion and with a reduced dependence on micellar incorporation (Christensen et al., 1989; Çenesiz and Çiftci, 2020). For example, this pattern is clearly illustrated by the contrasting absorption kinetics of lauric acid (C12:0) and stearic acid (C18:0). Considering total absorption at the distal ileum, lauric acid, the main MCFA in these oils, showed higher digestibility (89–95%) than stearic acid, a predominant LCSFA with low digestibility (75–83%). In addition, more than 90% of lauric acid was absorbed in the jejunum, whereas stearic acid exhibited a more distributed absorption profile between the jejunum (60–70%) and the ileum (30–40%). This difference reflects their distinct absorption sites along the gastrointestinal tract, indicating a slower uptake and a more prolonged absorption process for LCSFA.

Interestingly, myristic acid (C14:0), although classified as an LCSFA, showed digestibility values comparable to MCFA such as lauric acid. This observation supports previous findings indicating that FA up to 14 carbon atoms may behave similarly to unsaturated FA in terms of absorption (Wiseman and Blanch, 1994; Zeitz et al., 2015), suggesting that its nutritional classification in broiler nutrition should consider functional behavior and therefore warrants future investigation.

When comparing fat sources, SO showed the highest digestibility of PUFA across all intestinal segments, consistent with its high degree of unsaturation and its ability to promote micelle formation and enhance lipid absorption (Krogdahl, 1985). However, the substitution of SO with MCFA-rich sources low in FFA content, such as BSFO and PKO, did not compromise LCSFA and MUFA utilization, while significantly increasing MCFA absorption. Although PUFA digestibility decreased in these treatments, this reduction was compensated by the higher absorption of MCFA, particularly lauric acid (C12:0), which reached digestibility values comparable to those observed for linoleic acid (C18:2 n-6) in birds fed the SO diet. These findings highlight that the commonly accepted negative association between saturation and digestibility is incomplete, as saturated fats rich in MCFA can achieve FA absorption efficiencies comparable to PUFA-rich sources, suggesting compensatory effects among FA classes ultimately sustain total fat utilization.

In contrast, birds fed the PKFAD diet, which is rich in FFA, consistently exhibited lower digestibility coefficients across most FA classes. Compared with the SO treatment, PKFAD reduced the digestibility of MUFA, PUFA, and individual saturated FA such as C16:0 at the distal ileum, whereas MCFA digestibility remained higher. More importantly, when compared with PKO, despite their similar FA composition but lower FFA content, PKFAD showed reduced digestibility of TFA, MCFA, and LCSFA, highlighting that elevated FFA levels in MCFA-rich diets impair fat digestion and, consequently, reduce dietary AME during the grower–finisher period. In line with the results obtained during the starter phase (Espinosa-de-los-Monteros-Peñafiel et al., 2026), these findings indicate that this detrimental effect is not limited to early development but extends into later growth.

The detrimental impact of high dietary FFA levels observed in the present study is consistent with previous reports (Wiseman et al., 1991; Leeson and Atteh, 1995; Rodriguez-Sanchez et al., 2019b). Elevated FFA concentrations reduce the availability of monoacylglycerols required for mixed micelle formation and promote the formation of insoluble soaps with divalent cations, thereby limiting FA absorption. Notably, the negative effects of FFA were not restricted to LCSFA, as MCFA and MUFA digestibility was also reduced in PKFAD-fed birds, indicating a general impairment of lipid digestion processes. This contrasts with some studies in grower–finisher broilers reporting limited effects of moderate FFA levels (< 35%) (Rodriguez-Sanchez et al., 2021) and suggests that the very high FFA inclusion level in PKFAD exceeded the capacity of the digestive system to compensate, even at later stages of development. The analysis of the relative contribution of intestinal segments supports these findings. The jejunum accounted for the majority of FA absorption, and compared with starter birds, grower–finisher broilers exhibited a higher proportion of jejunal absorption, as previously described (Tancharoenrat et al., 2014; Rodriguez-Sanchez et al., 2019a; Espinosa-de-los-Monteros-Peñafiel et al., 2026). This age-related increment in proximal absorption suggests a more efficient and rapid overall lipid digestion process as the gastrointestinal tract matures. Specifically, MCFA absorption occurred almost entirely in the jejunum, with the ileum contributing less than 4% in broilers fed MCFA-rich diets, indicating that MCFA are not only highly digestible but also rapidly absorbed in the proximal segments of the intestine. In contrast, LCSFA exhibited a greater relative ileal contribution (9-19%), confirming a slower absorption rate. In birds fed the PKFAD diet, a shift toward increased ileal contribution was observed across most FA classes, indicating a delayed absorption process likely driven by impaired micellar formation in the proximal intestine (Jiménez-Moya et al., 2021a).

### Histomorphology of jejunum and ileum

The present study hypothesized that inclusion of MCFA-rich oils would improve intestinal morphology compared with SO. Jejunal morphology was not affected by dietary fat source, as Vh, Cd, and the Vh:Cd ratio did not differ among treatments (*P* > 0.05), indicating that none of the evaluated fat sources impaired mucosal integrity in this intestinal segment. The Vh:Cd ratio, widely recognized as an indicator of intestinal functionality and absorptive capacity (Pluske et al., 1996; Awad et al., 2009), remained unchanged across treatments. Similarly, in the distal ileum, neither Cd nor the Vh:Cd ratio was affected by dietary treatment, suggesting that the structural balance between absorptive surface and epithelial renewal remained largely unchanged in this intestinal segment. These findings are consistent with those reported during the starter phase (Espinosa-de-los-Monteros-Peñafiel et al., 2026). In contrast, birds fed the PKO-diet exhibited lower Vh than those fed SO-diet (1,082 vs. 1,266 μm; *P =* 0.040). Reduced Vh is generally associated with a decrease in the absorptive surface area, and may therefore suggest a lower absorptive capacity. However, broilers fed PKO did not differ from SO-fed birds in TFA digestibility or growth performance. Therefore, the lower ileal Vh is more likely to reflect an adaptative response to the dietary fat source rather than a negative effect on intestinal functionality.

### Microbiological counting

The intestinal microbiota plays a key role in nutrient utilization and host defense against pathogenic bacteria (Liu et al., 2021). In the present study, dietary oils source selectively influenced *Lactobacillus* populations in both the distal jejunum and ileum, whereas no effects were observed for *Enterobacteriaceae*, total coliforms, *Escherichia coli*, or the *Lactobacillus:Enterobacteriaceae* ratio. These results suggest that the dietary fat source did not disrupt the overall microbial balance, in agreement with the findings reported during the starter period (Espinosa-de-los-Monteros-Peñafiel et al., 2026). Broilers fed BSFO and PKO maintained *Lactobacillus* counts comparable to those observed in the SO group, which contrasts with the findings of Kierończyk et al. (2026), who reported a reduction in some *Lactobacillus* genus abundance following BSFO supplementation (2.5% and 5%) in jejunum. Contrary to what was observed for PKO and BSFO, PKFAD reduced *Lactobacillus* counts in a segment-dependent manner, showing lower populations in the distal jejunum compared to SO and PKO, and in the distal ileum relative to BSFO and PKO. This response may reflect differences in luminal conditions along the intestinal tract. Elevated dietary FFA levels are known to impair fat digestibility and increase the amount of unabsorbed fat reaching the distal intestine (Sibbald and Kramer, 1980; Wiseman et al., 1991; Kankaanpää et al., 2001), potentially creating conditions less favorable for *Lactobacillus* colonization. Accordingly, the lower *Lactobacillus* counts observed in PKFAD-fed broilers may be associated with the high FFA and LCSFA content of this oil, which reduced fat digestibility and altered the intestinal luminal environment. In the present study, MCFA-rich oils in TAG form supported *Lactobacillus* populations comparable to those observed with a conventional vegetable oil, whereas the FFA-rich PKFAD resulted in lower *Lactobacillus* counts without affecting the overall microbial balance, as indicated by the unchanged *Lactobacillus*:Enterobacteriaceae ratio.

### Cecal VFA content

Cecal short-chain fatty acids (**SCFA**) are important microbial metabolites involved in gut health and energy supply for the host (Lan et al., 2005). In the present study, total SCFA concentrations were not affected by dietary oil sources, and no significant differences were detected in the proportions of acetate, butyrate, valerate, or branched-chain fatty acids, indicating that cecal fermentation remained stable across treatments. Among individual SCFA, propionate tended to be higher in SO and BSFO-fed broilers compared with PKO and PKFAD groups (*P =* 0.063). As propionate is primarily produced by *Bacteroidetes* spp. through the succinate pathway (Rivière et al., 2016; Begunova et al., 2019), this tendency may reflect subtle diet-related shifts in microbial community composition rather than a direct effect of dietary MCFA content (Jadhav et al., 2025). The overall stability of cecal fermentation parameters, also reported during the starter phase (Espinosa-de-los-Monteros-Peñafiel et al., 2026), is consistent with the proximal absorption of MCFA in broilers. At the 6% inclusion level used in this study, MCFA were efficiently absorbed in the proximal intestine, as confirmed by the high digestibility values observed at the distal jejunum and ileum, and would therefore not be expected to reach the cecum in sufficient amounts to substantially influence fermentation activity (Zentek et al., 2011; Gomez-Osorio et al., 2021). Taken together, these results indicate that inclusion of MCFA-rich oil sources at 6% does not markedly disrupt cecal fermentation in grower-finisher broilers.

### Fatty acid composition of abdominal fat pad

In the present study, the inclusion of MCFA-rich oils did not modify the relative weight of the AFP in grower–finisher broilers, in agreement with Kim et al. (2020) and Wongsuthavas et al. (2026). In contrast, Wang et al. (2015) reported a reduction in AFP relative weight when SO was replaced by coconut oil. This response is consistent with the metabolic fate of MCFA, which, after intestinal absorption, are preferentially transported via the portal vein to the liver and rapidly oxidized through β-oxidation, thereby limiting their availability for re-esterification and storage in adipose tissue (Bach et al., 1996; Ee et al., 2024). This metabolic behavior contrasts with that of dietary LCSFA, which have been associated with increased fat deposition in broilers (Sanz et al., 2000; Crespo and Esteve-García, 2002; Vilarrasa et al., 2015). In particular, broilers fed palm oil showed higher abdominal fat than those supplemented with black soldier fly larvae oil–calcium salt (Aprianto et al., 2023). The FA profile of the AFP reflected the dietary FA supply, consistent with previous observations in broiler chickens (Sanz, 1999; Crespo and Esteve-García, 2001). However, tissue FA composition is also shaped by endogenous lipid metabolism, as SFA and MUFA may originate either from direct dietary incorporation or from *de novo* synthesis, which involves elongation and desaturation processes. Accordingly, the higher deposition of LCSFA and MUFA in broilers fed lauric acid-rich diets likely reflects, at least in part, the metabolic conversion of dietary lauric acid provided at high levels. In contrast, PUFA, being primarily of dietary origin, were deposited at higher proportions in SO-fed broilers compared with those fed the MCFA-rich oils. As a result, broilers fed MCFA-rich diets showed a more saturated AFP FA profile, as reflected by lower UFA/SFA ratios than SO-fed broilers. These findings are consistent with previous reports in which dietary inclusion of BSFO or coconut oil increased the proportion of C12:0 and reduced PUFA deposition in broiler AFP (Kim et al., 2020) and breast meat (Schiavone et al., 2017).

Among the MCFA-rich treatments, broilers fed insect oil (BSFO) showed the highest UFA/SFA ratio in the AFP, which can be attributed to the higher PUFA and lower MCFA content of this diet compared with palm kernel-derived diets. Similar results were reported by Kim et al. (2020), who observed higher PUFA content in the AFP of broilers fed BSFO compared with coconut oil. These results indicate that differences among MCFA-rich lipid sources are also influenced by their overall FA composition. Regarding the effect of FFA, broilers fed PKFAD, an oil characterized by a FA profile similar to PKO but a markedly higher FFA level, showed higher LCSFA and lower lauric acid (C12:0) deposition in the AFP compared with PKO. This response appears primarily driven by the higher dietary LCSFA concentration in PKFAD rather than by its FFA content. To the authors' knowledge, studies directly comparing PKO and PKFAD in broilers with data on AFP composition are scarce, limiting direct comparisons with the published literature. In terms of carcass quality, diet-induced changes in AFP unsaturation may be relevant given their potential effects on fat physical properties and oxidative stability (Sanz, 1999). However, the nutritional implications for human consumption of the reduced UFA/SFA ratio in adipose tissue observed in broilers fed the MCFA-rich diets warrant further investigation. Overall, these results indicate that replacing SO with MCFA-rich oils in grower–finisher broiler diets does not modify AFP weight but modifies its FA composition toward lower PUFA and higher SFA (both LCSFA and MCFA) resulting in a lower UFA/SFA ratio.

In conclusion, this study supports the use of MCFA-rich oils with low FFA content as viable alternatives to conventional oil sources in grower–finisher broiler diets, particularly regarding fat digestibility and feed efficiency. Medium-chain fatty acids exhibited consistently high ileal digestibility (90–96%), especially lauric acid (C12:0), which was predominantly absorbed in the jejunum, accounting for more than 90% of total absorption. In older broilers, the efficiency of MCFA utilization was comparable to, and in some cases greater than, that of PUFA, and clearly superior to that of LCSFA, such as palmitic and stearic acids. These results indicate that the inclusion of crude MCFA-rich oils, particularly those rich in C12:0 and low in FFA, such as BSFO and PKO, support efficient fat digestion while maintaining growth performance comparable to SO. In contrast, the high FFA content of PKFAD impaired FA digestibility, particularly MCFA utilization, reduced dietary AME, and compromised final BW, highlighting the importance of fat quality in grower-finisher nutrition. No major differences were observed in intestinal morphology; however, broilers fed PKFAD showed reduced *Lactobacillus* populations. Despite this, the *Lactobacillus*:Enterobacteriaceae ratio was not affected. Replacing SO with MCFA-rich oils did not affect AFP weight but modified its FA composition, resulting in lower PUFA content, higher SFA (including both LCSFA and MCFA) and a reduced UFA/SFA ratio. Overall, MCFA-rich fat sources supplied in TAG form represent viable alternative lipid ingredients for grower–finisher broiler diets, contributing to diversification of fat sources and reduced reliance on conventional vegetable oils. Their use may also enhance the valorization of agro-industrial lipid by‑products, aligning with circular and resource-efficient strategies in poultry production. In addition, the efficient absorption of fatty acids such as C12:0 at this stage highlights their relevance as readily available energy sources in grower–finisher broiler nutrition.

## Author contributions

M. Espinosa-de-los-Monteros-Peñafiel contributed substantially to the acquisition, curation, analysis, and interpretation of data, as well as to the development of the study methodology and data visualization. He drafted the manuscript and participated in its critical revision. He reviewed and approved the final version of the manuscript for publication. He agrees to be accountable for all aspects of the work and to ensure that any questions related to the accuracy or integrity of the study are appropriately investigated and resolved.

E. Llauradó-Calero contributed substantially to data curation, formal data analysis, and interpretation of the results. He contributed to drafting the manuscript and critically revised it for important intellectual content. He reviewed and approved the final version of the manuscript for publication. He agrees to be accountable for all aspects of the work and to ensure that any questions related to the accuracy or integrity of the study are appropriately investigated and resolved.

L. Castillejos contributed substantially to the conception and design of the study, methodology development, investigation, interpretation of results, resource provision, and supervision of the research. She critically reviewed the manuscript and contributed to the refinement of its scientific content. She reviewed and approved the final version of the manuscript for publication. She agrees to be accountable for all aspects of the work and to ensure that any questions related to the accuracy or integrity of the study are appropriately investigated and resolved.

R. Sala contributed substantially to the conception and design of the study, methodology development, investigation, project administration, acquisition of funding, resource provision, supervision, and interpretation of results. She provided critical revisions to the manuscript and contributed important intellectual insights. She reviewed and approved the final version of the manuscript for publication. She agrees to be accountable for all aspects of the work and to ensure that any questions related to the accuracy or integrity of the study are appropriately investigated and resolved.

A. Tres contributed substantially to the development of the study methodology and to the interpretation of the findings. She critically reviewed the manuscript and provided intellectual input that strengthened its scientific content. She reviewed and approved the final version of the manuscript for publication. She agrees to be accountable for all aspects of the work and to ensure that any questions related to the accuracy or integrity of the study are appropriately investigated and resolved.

M.D. Soler contributed substantially to methodology development and to the interpretation of the study results. She critically evaluated the manuscript and contributed important revisions to its intellectual content. She reviewed and approved the final version of the manuscript for publication. She agrees to be accountable for all aspects of the work and to ensure that any questions related to the accuracy or integrity of the study are appropriately investigated and resolved.

A.C. Barroeta contributed substantially to the conception and design of the study, data curation, formal analysis, investigation, methodology development, project administration, acquisition of funding, resource provision, supervision, and interpretation of results. She critically reviewed the manuscript throughout its preparation and contributed significantly to its intellectual development. She reviewed and approved the final version of the manuscript for publication. She agrees to be accountable for all aspects of the work and to ensure that any questions related to the accuracy or integrity of the study are appropriately investigated and resolved.

## Disclosures

The authors declare the following financial interests/personal relationships which may be considered as potential competing interests:

Ana Cristina Barroeta reports financial support was provided by Spain Ministry of Science Innovation and Universities. If there are other authors, they declare that they have no known competing financial interests or personal relationships that could have appeared to influence the work reported in this paper.

## References

[bib0001] AOAC International (2023).

[bib0002] Aprianto M.A., Kurniawati A., Hanim C., Ariyadi B., Al Anas M. (2023). Effect supplementation of black soldier fly larvae oil (*Hermetia illucens* L.) calcium salt on performance, blood biochemical profile, carcass characteristic, meat quality, and gene expression in fat metabolism broilers. Poult. Sci..

[bib0003] Aviagen (2022).

[bib0004] Awad W.A., Ghareeb K., Abdel-Raheem S., Böhm J. (2009). Effects of dietary inclusion of probiotic and synbiotic on growth performance, organ weights, and intestinal histomorphology of broiler chickens. Poult. Sci..

[bib0005] Bach A.C., Ingenbleek Y., Frey A. (1996). The usefulness of dietary medium-chain triglycerides in body weight control: fact or fancy?. J. Lipid Res..

[bib0006] Baltić B., Ćirić J., Šefer D., Radovanović A., Đorđević J., Glišić M., Bošković M., Baltić M.Ž., Đorđević V., Marković R. (2018). Effect of dietary supplementation with medium chain fatty acids on growth performance, intestinal histomorphology, lipid profile and intestinal microflora of broiler chickens. South Afr. J. Anim. Sci..

[bib0007] Begunova A.V., Rozhkova I.V., Zvereva E.A., Glazunova O.A., Fedorova T.V. (2019). Lactic and propionic acid bacteria: the formation of a community for the production of functional products with bifidogenic and hypotensitive properties. Appl. Biochem. Microbiol..

[bib0008] Carrapiso A.I., Timón M.L., Petrón M.J., Tejeda J.F., García C. (2000). In situ transesterification of fatty acids from Iberian pig subcutaneous adipose tissue. Meat Sci..

[bib0009] Çenesiz A.A., Çiftci İ. (2020). Modulatory effects of medium chain fatty acids in poultry nutrition and health. Worlds Poult. Sci. J..

[bib0010] Chen X., Jin J., Hou F., Song B., Li Z., Zhao Y. (2022). Effects of black soldier fly larvae oil on growth performance, immunity and antioxidant capacity, and intestinal function and microbiota of broilers. J. Appl. Poult. Res..

[bib0011] Christensen E., Hagve T.-A., Grønn M., Christophersen B.O. (1989). β-oxidation of medium chain (C8-C14) fatty acids studied in isolated liver cells. Biochim. Biophys. Acta BBA - Lipids Lipid Metab..

[bib0012] Cortinas L., Villaverde C., Galobart J., Baucells M.D., Codony R., Barroeta A.C. (2004). Fatty acid content in chicken thigh and breast as affected by dietary polyunsaturation level. Poult. Sci..

[bib0013] Crespo N., Esteve-García E. (2001). Dietary fatty acid profile modifies abdominal fat deposition in broiler chickens. Poult. Sci..

[bib0014] Crespo N., Esteve-García E. (2002). Dietary polyunsaturated fatty acids decrease fat deposition in separable fat depots but not in the remainder carcass. Poult. Sci..

[bib0015] Ee H.W., Ramiah S.K., Mookiah S., Idrus Z. (2024). Effects of medium-chain fatty acids on growth performance, microbial attributes, and fat deposition in broiler chicken. Czech J. Anim. Sci..

[bib0016] Espinosa-de-los-Monteros-Peñafiel M., Castillejos L., Sala R., Tres A., Soler M.D., Barroeta A.C. (2026). Medium-chain fatty acid-rich oils with varying free fatty acid levels as alternatives to soybean oil in broiler starter diets. Poult. Sci..

[bib0017] FEDNA. 2018. Necesidades nutricionales para avicultura: normas FEDNA. 2a ed.

[bib0018] Ferrini G., Baucells M.D., Esteve-García E., Barroeta A.C. (2008). Dietary polyunsaturated fat reduces skin fat as well as abdominal fat in broiler chickens. Poult. Sci..

[bib0019] Gomez-Osorio L.-M., Yepes-Medina V., Ballou A., Parini M., Angel R. (2021). Short and medium chain fatty acids and their derivatives as a natural strategy in the control of necrotic enteritis and microbial homeostasis in broiler chickens. Front. Vet. Sci..

[bib0020] Jadhav V.V., Fasina Y.O., Harrison S.H. (2025). Dietary polyunsaturated fatty acids effect on cecal microbiome profile of maturing broiler chicken. Poult. Sci..

[bib0021] Jiménez-Moya B., Barroeta A.C., Guardiola F., Soler M.D., Rodriguez-Sanchez R., Sala R. (2021). Replacement of palm oil with soybean acid oil in broiler chicken diet: fat digestibility and lipid class content along the intestinal tract. Animals.

[bib0022] Jiménez-Moya B., Barroeta A.C., Tres A., Soler M.D., Sala R. (2021). Soybean oil replacement by palm fatty acid distillate in broiler chicken diets: fat digestibility and lipid-class content along the intestinal tract. Animals.

[bib0023] Kankaanpää P.E., Salminen S.J., Isolauri E., Lee Y.K. (2001). The influence of polyunsaturated fatty acids on probiotic growth and adhesion. FEMS Microbiol. Lett..

[bib0024] Kierończyk B., Drażbo A., Juśkiewicz J., Kozłowski K., Szymkowiak P., Ciesielska L., Praska D., Aslam M.R., Rawski M., Józefiak D. (2026). Evaluation of the jejunal microbiota and microbial markers in laying hens fed diets with black soldier fly larval fat as a partial or total replacement for soybean oil. Poult. Sci..

[bib0025] Kim B., Bang H.T., Kim K.H., Kim M.J., Jeong J.Y., Chun J.L., Ji S.Y. (2020). Evaluation of black soldier fly larvae oil as a dietary fat source in broiler chicken diets. J. Anim. Sci. Technol..

[bib0026] Krogdahl Å. (1985). Digestion and absorption of lipids in poultry. J. Nutr..

[bib0027] Lan Y., Verstegen M.W.A., Tamminga S., Williams B.A. (2005). The role of the commensal gut microbial community in broiler chickens. Worlds Poult. Sci. J..

[bib0028] Leeson S., Atteh J.O. (1995). Utilization of fats and fatty acids by turkey poults. Poult. Sci..

[bib0029] Liu L., Li Q., Yang Y., Guo A. (2021). Biological function of short-chain fatty acids and its regulation on intestinal health of poultry. Front. Vet. Sci..

[bib0030] Pluske J.R., Thompson M.J., Atwood C.S., Bird P.H., Williams I.H., Hartmann P.E. (1996). Maintenance of villus height and crypt depth, and enhancement of disaccharide digestion and monosaccharide absorption, in piglets fed on cows' whole milk after weaning. Br. J. Nutr..

[bib0031] Ravindran V., Tancharoenrat P., Zaefarian F., Ravindran G. (2016). Fats in poultry nutrition: digestive physiology and factors influencing their utilisation. Anim. Feed Sci. Technol..

[bib0032] Rivière A., Selak M., Lantin D., Leroy F., De Vuyst L. (2016). Bifidobacteria and butyrate-producing colon bacteria: importance and strategies for their stimulation in the human gut. Front. Microbiol..

[bib0034] Rodriguez-Sanchez R., Tres A., Sala R., Garcés-Narro C., Guardiola F., Gasa J., Barroeta A.C. (2019). Effects of dietary free fatty-acid content and saturation degree on lipid-class composition and fatty-acid digestibility along the gastrointestinal tract in broiler starter chickens. Poult. Sci..

[bib0033] Rodriguez-Sanchez R., Tres A., Sala R., Guardiola F., Barroeta A.C. (2019). Evolution of lipid classes and fatty acid digestibility along the gastrointestinal tract of broiler chickens fed different fat sources at different ages. Poult. Sci..

[bib0035] Rodriguez-Sanchez R., Tres A., Sala R., Soler M.D., Guardiola F., Barroeta A.C. (2021). Effects of free-fatty-acid content and saturation degree of the dietary oil sources on lipid-class content and fatty-acid digestibility along the gastrointestinal tract in broilers from 22 to 37 days of age. Poult. Sci..

[bib0036] Sanz M. (1999). Higher lipid accumulation in broilers fed on saturated fats than in those fed on unsaturated fats. Br. Poult. Sci..

[bib0037] Sanz M., Lopez-Bote C.J., Menoyo D., Bautista J.M. (2000). Abdominal fat deposition and fatty acid synthesis are lower and β-oxidation is higher in broiler chickens fed diets containing unsaturated rather than saturated fat. J. Nutr..

[bib0038] Schiavone A., Cullere M., De Marco M., Meneguz M., Biasato I., Bergagna S., Dezzutto D., Gai F., Dabbou S., Gasco L., Dalle Zotte A. (2017). Partial or total replacement of soybean oil by black soldier fly larvae (*Hermetia illucens* L.) fat in broiler diets: effect on growth performances, feed-choice, blood traits, carcass characteristics and meat quality. Ital. J. Anim. Sci..

[bib0039] Sibbald I.R., Kramer J.K.G. (1980). The effect of the basal diet on the utilization of fat as a source of true metabolizable energy, lipid, and fatty acids. Poult. Sci..

[bib0040] Sukhija P.S., Palmquist D.L. (1988). Rapid method for determination of total fatty acid content and composition of feedstuffs and feces. J. Agric. Food Chem..

[bib0041] Szabó R.T., Kovács-Weber M., Zimborán Á., Kovács L., Erdélyi M. (2023). Effects of short- and medium-chain fatty acids on production, meat quality, and microbial attributes—a review. Molecules.

[bib0042] Tancharoenrat P., Ravindran V., Zaefarian F., Ravindran G. (2014). Digestion of fat and fatty acids along the gastrointestinal tract of broiler chickens. Poult. Sci..

[bib0043] Varona E., Tres A., Rafecas M., Vichi S., Barroeta A.C., Guardiola F. (2021). Composition and nutritional value of acid oils and fatty acid distillates used in animal feeding. Animals.

[bib0044] Varona E., Tres A., Rafecas M., Vichi S., Barroeta A.C., Guardiola F. (2021). Methods to determine the quality of acid oils and fatty acid distillates used in animal feeding. MethodsX.

[bib0045] Vilarrasa E., Codony R., Esteve-García E., Barroeta A.C. (2015). Use of re-esterified oils, differing in their degree of saturation and molecular structure, in broiler chicken diets. Poult. Sci..

[bib0046] Villaverde C., Baucells M.D., Cortinas L., Barroeta A.C. (2006). Effects of dietary concentration and degree of polyunsaturation of dietary fat on endogenous synthesis and deposition of fatty acids in chickens. Br. Poult. Sci..

[bib0047] Wang J., Wang X., Li J., Chen Y., Yang W., Zhang L. (2015). Effects of dietary coconut oil as a medium-chain fatty acid source on performance, carcass composition and serum lipids in male broilers. Asian-Australas. J. Anim. Sci..

[bib0049] Wiseman J., Blanch A. (1994). The effect of free fatty acid content on the apparent metabolisable energy of coconut/palm kernel oil for broiler chickens aged 12 and 52 days. Anim. Feed Sci. Technol..

[bib0050] Wiseman J., Powles J., Salvador F. (1998). Comparison between pigs and poultry in the prediction of the dietary energy value of fats. Anim. Feed Sci. Technol..

[bib0051] Wiseman J., Salvador F. (1991). The Influence of free fatty acid content and degree of saturation on the apparent metabolizable energy value of fats fed to broilers. Poult. Sci..

[bib0052] Wiseman J., Salvador F., Craigon J. (1991). Prediction of the apparent metabolizable energy content of fats fed to broiler chickens. Poult. Sci..

[bib0048] Wongsuthavas S., Wanapat M., Yuangklang C., Vasupen K., Wongtrairat W., Khonkhaeng B., Khotsakdee J., Beynen A.C. (2026). Dietary fat type modulates fatty acid digestibility, de novo lipogenesis, and energy expenditure in broiler chickens. Poult. Sci..

[bib0053] Xu C.J., Liu Q., Xu M., Ayalew H., Iqbal W., Lin J., Song J., Jin L.X., Song Z., Zhang H.J. (2025). Dietary medium-chain fatty acids mitigate Escherichia coli O78 infection in broilers by enhancing intestinal immune barrier and modulating microbiota. Poult. Sci..

[bib0054] Zeitz J.O., Fennhoff J., Kluge H., Stangl G.I., Eder K. (2015). Effects of dietary fats rich in lauric and myristic acid on performance, intestinal morphology, gut microbes, and meat quality in broilers. Poult. Sci..

[bib0055] Zentek J., Buchheit-Renko S., Ferrara F., Vahjen W., Kessel A.G.V., Pieper R. (2011). Nutritional and physiological role of medium-chain triglycerides and medium-chain fatty acids in piglets. Anim. Health Res. Rev..

